# Robust Wireless Sensor and Actuator Networks for Networked Control Systems

**DOI:** 10.3390/s19071535

**Published:** 2019-03-29

**Authors:** Bongsang Park, Junghyo Nah, Jang-Young Choi, Ick-Jae Yoon, Pangun Park

**Affiliations:** 1 Department of Radio and Information Communications Engineering, Chungnam National University, Daejeon 34134, Korea; bong4ang@cnu.ac.kr; 2 Department of Electrical Engineering, Chungnam National University, Daejeon 34134, Korea; jnah@cnu.ac.kr (J.N.); choi_jy@cnu.ac.kr (J.-Y.C.); ijyoon@cnu.ac.kr (I.-J.Y.)

**Keywords:** joint design, wireless networks, clustering, scheduling, robustness, wireless networked control systems

## Abstract

The stability guarantee of wireless networked control systems is still challenging due to the complex interaction among the layers and the vulnerability to network faults, such as link and node failures. In this paper, we propose a robust wireless sensor and actuator network (R-WSAN) to maintain the control stability of multiple plants over the spatial-temporal changes of wireless networks. The proposed joint design protocol combines the distributed controller of control systems and the clustering, resource scheduling, and control task sharing scheme of wireless networks over a hierarchical cluster-based network. In particular, R-WSAN decouples the tasks from the inherently unreliable nodes and allows control tasks to share between nodes of wireless networks. Our simulations demonstrate that R-WSAN provides the enhanced resilience to the network faults for sensing and actuation without significantly disrupting the control performance.

## 1. Introduction

Embedded wireless sensor and actuator networks (WSAN) are becoming a fundamental network infrastructure to monitor and operate the safety-critical automation systems since they provide many benefits, such as low installation and maintenance costs in adversarial environments [1,2,3,4]. WSANs are particularly appealing for various control applications such as factory automation, power systems automation, and mining industry [3]. Several international organizations such as international society of automation (ISA) [5] and highway addressable remote transducer protocol (HART) [6] have been supporting wireless networks for industrial control systems. In wireless networked control systems (WNCS), sensors transmit their measurements of the physical plant to the controller over a wireless network. Controllers compute the control signal based on the received sensing information and forward it to the actuators.

To realize WSANs for the closed-loop control, WNCS have received considerable attention in recent research on both control and communication societies [1,7]. WNCSs present a conceptual shift of focus away from a passive information gathering viewpoint of traditional sensor networks, to one of the closed-loop control of the physical plants. Despite its success of both the control system and the wireless network, much of the literature separately considers the design problem of WNCSs. In fact, there are few studies on WNCSs that jointly consider the control design and the wireless network under realistic wireless conditions [7,8]. The main challenges to design and analyze WNCSs are as follows.

1. Recent low-power mesh networks provide loss rates in the range of one percent [9,10,11]. Even though the high end-to-end reliability possibly supports the closed-loop control systems of the slow dynamical systems, the control stability not only depends on the reliability, but also the traffic generation interval and delay [1]. The complex interactions of the reliability, delay, and traffic load leads to difficult problems even in simple WNCS scenarios [12]. For instance, the retransmissions improve the reliability at the cost of increasing jitter performance. The jitter is difficult to compensate for, especially, if the delay variability is large in the control systems. Furthermore, outdated packets are not generally useful for critical control applications [13].
2. The wireless networks are inherently exposed to network faults such as link and node failures. The network must detect and repair the faults over a lossy network since the control algorithm is not strong enough to guarantee the deterministic robustness of WNCS. In fact, the fundamental problem is due to the node level programming of WSANs since the set of control tasks is associated with the unreliable embedded node [14]. This is one of the key reasons of the lack of robustness in WNCSs.
3. Since the computing resources on embedded nodes are limited [1], the calculations necessary to implement the protocol must be computationally light. Furthermore, the protocol should be scalable to the large network since the number of embedded sensors is significantly increasing due to the evolution of the microelectromechanical systems and the computing hardware [1]. Scalability means the efficient load balancing and network resource management to guarantee the network robustness in this paper. In addition, the tractable analytical model of the network is quite useful for the overall control system design.

To achieve the robust WNCSs, there is a strong need to rethink the wireless network design for the reliable closed-loop control [15]. Current approaches for NCSs generally rely on a minimal set of reliability and delay requirements based on unrealistic assumptions of wireless networks [16,17]. However, this does not guarantee the stability of control systems in practice.

This paper focuses on the joint design problem of WNCSs to systematically explore the interactions between control systems and wireless networks. We propose a robust wireless sensor and actuator network (R-WSAN) to maintain the control stability of multiple plants over a hierarchical cluster-based network. By considering the control stability requirement, R-WSAN achieves the high robust performance by combining the distributed controller of control systems and the network clustering, resource scheduling, and control task sharing between nodes of wireless networks. In particular, the novel task allocation approach is proposed to bind the same control task to multiple nodes to support the fault-tolerant network. Our simulations demonstrate that R-WSAN provides the enhanced resilience to the network faults for sensing and actuation without significantly disrupting the control system performance. We show that even though it requires some network overhead, R-WSAN is practical to achieve the robust closed-loop control performance.

The remainder of this paper is organized as follows. Section 2 surveys related works. Section 3 describes the system model and states the main assumptions. Section 4 introduces the essential components of R-WSAN. In Section 5, we present more details of the network resource management of R-WSAN. Section 6 shows its effectiveness via simulations and compares it to the centralized approach. Section 7 gives concluding remarks and directions for future works.

## 2. Related Works

In this section, we discuss the related works on the clustering, the multi-hop scheduling, and the joint design for WNCSs.

Clustering of WSNs is well investigated in the previous literature since it provides the efficient data fusion with the low energy consumption [18]. The low-energy adaptive clustering hierarchy (LEACH) [19] is one of the first hierarchical cluster-based protocols for WSNs. In LEACH, the sensor nodes organize themselves into local clusters, with one node acting as the cluster head. LEACH [19] utilizes the adaptive clusters by rotating cluster heads to distribute the energy consumption among all the sensors. The cluster heads not only collect data from their clusters, but also aggregate the collected data to reduce the amount of messages to send to the central coordinator, which enhances the network lifetime. Many clustering algorithms of WSNs are inspired by LEACH [20,21,22].

The authors propose the randomized clustering algorithm based on the optimal values of joining parameters in [20]. Furthermore, it uses the multi-hop forwarding technique for intra-cluster and inter-cluster communications. In [21], the hierarchical structure is extended to multi-layers where cluster heads are rotated based on their residual energy and nodal degree. The effect of the multi-hop on the clustering protocol is investigated in [22].

While most clustering approaches consider the data aggregation or convergecast from the large number of sensors to the central coordinator, the clustering problem of our scenarios is different from traditional WSNs. WNCS generally consists of heterogeneous devices with sensors, actuators, and access points in the control domain. Even though the cluster head rotation is one of the major approaches to achieve the energy efficiency in the traditional WSN clustering, these approaches are not suitable for the industrial control context. The main reason is that sensors and actuators are located around control plants with its own time-critical functionality. In addition, the industrial wireless network has several access points and central manager with higher computation and communication capability to guarantee the reliable and timely communication [5,6]. Hence, the clustering problem of WNCSs must consider both the control system requirements and the network conditions.

The time synchronized channel hopping (TSCH) protocol is a promising MAC standard to achieve the reliable communications for the low-power critical applications [23]. The main idea of TSCH is to combine the time slotted operation and the channel hopping based on the time synchronization. However, the TSCH standard does not specify how to schedule and maintain the time slots and the channels [23].

In this context, the resource scheduling problem of the TSCH framework has received significant attention [24]. While the scheduling scheme can be either centralized or distributed, centralized schedules are generally better than distributed ones for the static networks [25,26]. Some centralized schedulers [27,28] consider the static network with predefined traffic patterns and achieve the extremely high reliable communications. Distributed solutions were also proposed, where a rendezvous slot is used for discovery and slot installation [29]. A conflict-aware real-time routing approach is proposed for WirelessHART networks [30]. The key approach of conflict-aware routing is to incorporate transmission conflicts and scheduling with its routing decisions in order to improve real-time performance. By incorporating conflict delays into the routing decisions, conflict-aware real-time routing algorithms allow a WSAN to accommodate more real-time flows while meeting their deadlines. Experiments on a physical testbed and in numerical simulations show that conflict-aware routing can lead to as much as a three-fold improvement in the real-time capacity of a WSAN. Recently, the Orchestra protocol is proposed to provide a simple schedule that nodes can maintain based on the local knowledge of their neighborhood for non-deterministic low-power networks [31]. The scheduler includes a set of dedicated slots dependent on MAC, routing, and application layers.

Some recent research of WNCSs investigated the joint design problem between wireless networks and control systems [1,8,32,33]. In [8], a wireless process control system is proposed by integrating the control design and the network routing of the WirelessHART standard [6]. The model predictive control design combines the observer based on an extended Kalman filter and the actuator buffer of recent control signals in order to mitigate the effect of packet loss in both sensing and actuating links, respectively. On the other hand, the network routing of WirelessHART proposes two routing strategies, namely, single-path source routing and multi-path graph routing. The experimental results show that the packet losses of the actuating link is more critical than the one of the sensing link. Hence, the paper suggests the source routing for sensing and the multi-path graph routing for actuation to improve the robustness of the control performance.

In [32], the cross-layer optimized control protocol is proposed for minimizing the worst-case performance loss of multiple control systems. The centralized proposed protocol is designed for a general wireless sensor and actuator network where both sensor-controller and controller-actuator connections are over a multi-hop mesh network. The design approach relies on a constrained max-min optimization problem, where the objective is to maximize the minimum resource redundancy of the network and the constraints are the stability of the closed-loop control systems and the schedulability of the communication resources. The stability condition of the control system has been formulated in the form of update deadline constraint. The optimal operation point of the protocol is automatically set in terms of the sampling period, slot scheduling, and routing, and is achieved by solving a linear programming problem, which adapts to system requirements and link conditions.

In [33], the sampling period optimization problem is formulated to minimize the control cost while ensuring end-to-end delay constraints for a multi-hop network. The linear quadratic cost function is used as the control performance measure, which is a function of the sampling period. Due to the complex interdependence of the decision variables, the heuristic solutions are obtained by using different algorithms such as a subgradient method, simulated annealing-based penalty method, greedy heuristic method, and approximated convex optimization method. The performance is then evaluated in terms of execution time and achieved control cost of WNCSs.

To the best of our knowledge, our paper is the first study formulating jointly communication and control performance to guarantee the stability of multiple plants over the hierarchical cluster-based network. In particular, we provide the overall robust performance of control stability combining the distributed controller of control systems and the clustering, scheduling, and network-level task sharing technique of wireless networks. Even though many optimization problems of control, scheduling, and routing have been proposed for WSNs, a very limited number of the joint framework has been proposed for WNCSs.

## 3. System Model and Assumption

**Figure 1:**
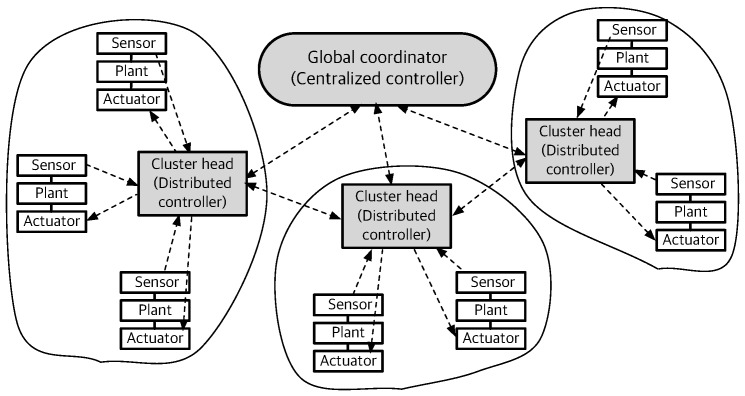
General structure of wireless networked control systems. Multiple plants are controlled by multiple controllers. A wireless network closes the loop from sensor to controller and from controller to actuator. The network includes sensors and actuators attached to the plants, cluster heads, and global coordinator.

Figure 1 depicts the system architecture of WNCSs where multiple plants are controlled over a wireless network. WNCS consists of several sensors and actuators attached to plants and controllers. Each sensor sends their measurements of the plant state to the their controllers. When the controller receives the plant states, actuation signals computed by the control algorithm are forwarded to actuators through the same WSAN. Wireless sensors transmit data in each assigned time slot dependent on the transmission scheduling scheme. Both the controller and actuator only respond to newly received data over unreliable wireless links. Hence, both the controller and actuator operate in an event-driven fashion, but each sensor operates in a time-driven fashion. Assuming sensors sample the plant state right before the transmission slot and transmit it during their allocated transmission time to the controller in order to minimize the delay.

Regarding the network aspects, the network consists of a global coordinator (GC), cluster heads (CHs), and a set of sensors and actuators. Please note that the wireless routers of WirelessHART or ISA100.11a standards can provide the functionalities of CHs.

We use the end devices to denote sensors or actuators. Assume that there are only a small number of CHs $N_{ch}$ with respect to several plants $N_{p}$, $N_{ch}<N_{p}$. A number of sensors $N_{s,i}$ and actuators $N_{a,i}$ are attached to plant *i*. Each link is described with its packet delivery rate (PDR).

We assume the heterogeneous radio transceiver and computing capabilities between GC, CH, and end device due to the different roles. GC and CH generally have the higher computation capability than the one of end devices. Hence, GC or CH can provide the controller task, while each end device only runs the dedicated time-critical task for sensing or actuation. All CHs and end devices are equipped with a half-duplex radio transceiver, implying that they cannot transmit and receive at the same time slot. On the other hand, GC supports the multi-channel transceiver.

## 4. Robust Wireless Sensor and Actuator Network

Our focus is on the design of WSANs to guarantee the control stability in the face of spatial-temporal network changes. R-WSAN instruments the network to adapt and reconfigure to changes while ensuring the control algorithm is within its stability constraints.

The control stability of R-WSAN is achieved through (i) efficient joint design of control and communication by abstracting the control stability requirement (ii) scalable network management through the clustering technique (iii) ultra-reliable network performance using the time and frequency diversity techniques (iv) low-cost fault-tolerant mechanism through the control task sharing between CHs.

### 4.1. Control Stability Requirement

In [1], we define three major metrics of WNCSs, namely, sampling interval, packet loss, and packet delay. Most works of control systems model the losses as prolongations of the sampling interval [34,35]. The reason is that a new packet is transmitted at the next transmission time with new data if a packet is dropped. Hence, both the controller and actuator observe the time-varying sampling interval even if the sensing and actuating links operate in a fixed time interval. The time-varying sampling interval of successfully received information called transmission interval (TI) effectively captures the essential characteristics of sampling interval, packet loss, and packet delay [35,36]. In NCS, the delays are generally assumed to be smaller than the transmission intervals. It implies that each transmitted packet arrives before the next sampling instance.

The uncertain time-varying TIs and time-varying delays provide the fundamental interactions between control and communication layers [34,35,37]. In the control community, much research has been conducted to analyze the stability of control systems for a given set of maximum allowable transmission interval (MATI) amd maximum allowable delay (MAD) values in [34,35,37]. Practical industrial control and automation systems set different communication performance requirements such as the cycle time, latency, scalability, and reliability level [3]. The cycle time and latency are strongly related to the MATI and MAD requirements of the control system analysis. The closed-loop system stability is guaranteed if the TI is smaller than MATI and the feedback communication delay (i.e., delay from the sensors to the assigned controller and from the controller to the actuators) along with the time needed for the control signal computation is less than MAD. Please note that the TI metric is generally more critical than the feedback delay for WNCS since it is a function of sampling interval, packet loss, and delay [38]. Furthermore, it is possible to minimize the feedback delay by tightly assigning sensing and actuating links of the plant, as we will discuss in Section 5.3.

Hence, we define the weight of plant *i* as

(1) $$\begin{array}{c} W_{i}=1-\frac{h_{i}}{\sum_{j=1}^{N_{p}}h_{j}} \end{array}$$

where $h_{i}$ is the MATI value of plant *i* and $N_{p}$ is the number of plants of the network. In Equation (1), the weight of plants increases, as the MATI requirement of control loops becomes smaller with respect to other plants.

To analyze the stability of control systems, linear matrix inequality conditions are verified on the polytopic overapproximation in [34,35,37]. The linear matrix inequality conditions are verified using the YALMIP [39] and the SeDuMi solver [40]. We use the analytical technique of the control stability in [37]. This technique effectively analyzes the stability to a given linear time-invariant plant model, a linear time-invariant controller model, and MATI and MAD bounds on the network uncertainties.

### 4.2. Hierarchical Cluster-Based Network

Clustering is particularly useful for control applications that require the analytical tractability and scalability to hundreds or thousands of nodes [18]. In R-WSAN, sensors and actuators communicate with controllers through a cluster-based multi-hop wireless network. Each cluster is a basic network topology presented in Figure 1, where all sensors contend to send their plant state to CH and CH transmits the control signal to each actuator. Each CH is able to run the control algorithm to efficiently handle the closed-loop system as a distributed controller. We consider a simple linear feedback control scheme. Since the controller must minimize the delay between sensing and actuating the plant to meet the stability constraint, sensing and actuating link associated with the same plant are allocated to the same cluster. This scheme is eminently suited for the closed-loop control applications.

R-WSAN separates the major network operations into GC and CH. GC forms the root of the hierarchy and supervises the entire network. It has responsible for configuring the network clustering, frame structure, managing routing tables, and communication scheduling between CH and GC. Due to the static nature of network, the cluster reconfiguration is not expected to change frequently. In case of long-term failure of nodes or links, GC reconfigures the network clusters. GC collects all plant state of the network to monitor machines for fault detection and diagnosis for control systems. Furthermore, based on the plant state information, GC may update the control gains or reference of some plants and transmit the new control gains to the corresponding CHs if it is needed.

On the other hand, CH basically configures the network resources such as communication scheduling between end device and CH. This achieves a large gain in the control performance and energy dissipation, as computation is much cheaper than communication. Furthermore, CH can aggregate the received sensing information and send it to the GC or an upper level CH in order to provide the entire plant states.

We limit the number of hops between end device and GC to 2. In a multi-hop network, increasing the number of retransmissions per hop may improve the end-to-end reliability, but at the risk of increasing network congestion and jitter and thus eventually leading to lossy and delayed control feedback [1].

### 4.3. Time and Channel Diversity

TSCH is a promising link solution since the closed-loop control generally has predefined periodic traffic patterns. By considering IEEE 802.15.4e [23] and ISA100.11a. [5], all data transmissions of R-WSAN are scheduled in dedicated time slots or shared time slots. Furthermore, concurrent transmissions are feasible to schedule on different channels. The time synchronization is triggered from GC down to end devices along the cluster-based network since all nodes must have the same sense of time. Each node updates the time synchronization based on a data or acknowledgement frame [23].

R-WSAN applies channel hopping to achieve the high robustness against jamming and interference from other wireless systems. We use a simple channel hopping method based on the global slot counter and channel offset comparable with TSCH [23]. We select the channel offset as a function of the unique CH identifier. Using this simple rule, each cluster can operate the contention-free channel hopping. Thus, all clusters can exchange their frames at the same slot using different channel offsets. Please note that all the nodes in the cluster compute the channel hopping sequence to transmit or receive without any extra negotiation. Even though we avoid the channel reuse to enhance the reliability and real-time performance, several practical algorithms have been proposed and applied in practice [41].

### 4.4. Control Task Share between Cluster Heads

**Figure 2:**
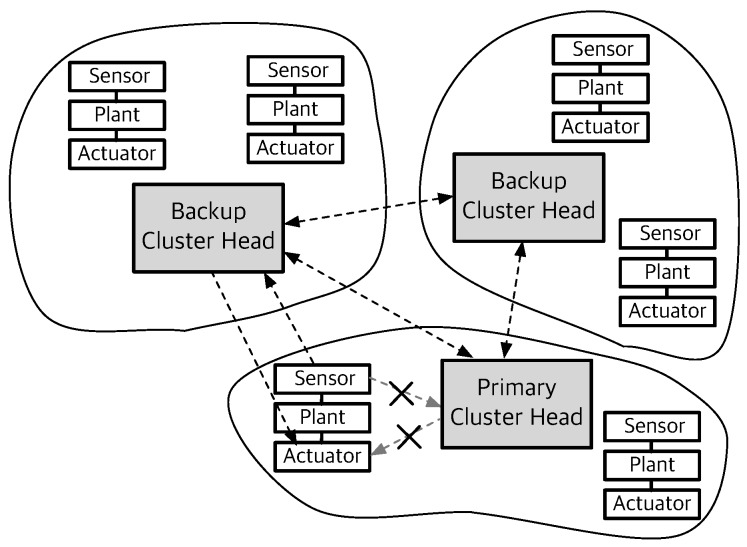
Control task sharing between cluster heads for critical control loops. The critical sensor accesses the backup cluster head through the multiple path. Each cluster head shares the critical sensing information with other cluster heads during the inter-cluster subframe.

The robust performance guarantee is hard to achieve in WNCSs since all network nodes and links can be faulty. One of our fundamental approaches is to bind the same control task to multiple CHs in order to support the fault-tolerant systems. We assign the control task (i.e., control algorithm) to the set of CHs, where each control task is assigned to a primary CH and other additional backup CHs, as shown in Figure 2. This technique increases system robustness to the link and CH failures by using different paths for sensing and actuating the plant.

In the industrial wireless standard, WirelessHART recommends that each node can use at least two separate paths to route data [6]. Thus, in R-WSAN, each end device has multiple CH candidates to deliver the plant state even though it belongs to the one of the cluster. Hence, each end device has the primary CH and backup CHs in its neighbor list. Multiple CHs can compute the control signal based on the received plant state and send it to the corresponding actuator if it can access it. Hence, the backup CHs support the multiple routing paths to improve the network robustness. We will provide more details in Section 5.4.

## 5. Network Resource Management

R-WSAN is a self-organizing, adaptive protocol that uses the clustering, time-frequency diversity, network-level task sharing to guarantee the control stability constraint of WNCSs. This section presents the essential components of the network resource management of R-WSAN, namely, (i) frame structure (ii) clustering (iii) scheduling (iv) task sharing.

### 5.1. Frame Structure

**Figure 3:**
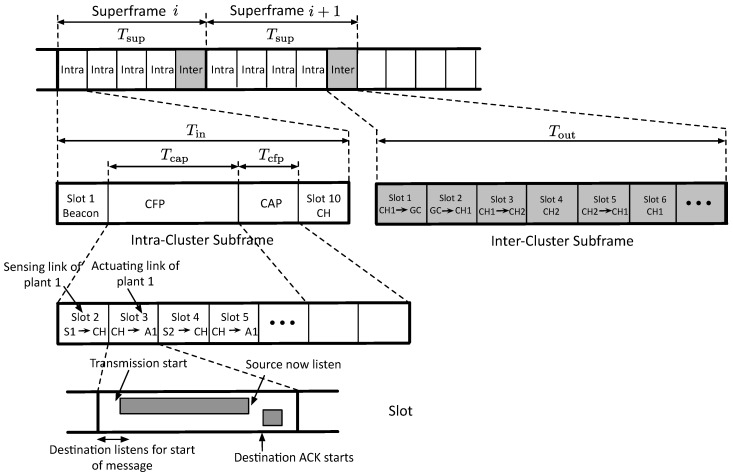
Frame structure of robust wireless sensor and actuator networks. Hierarchical time structure consisting with superframe, subframe, and time slot. Each intra-cluster subframe consists with a contention free period and a contention access period.

Figure 3 illustrates three time layers of the frame structure, namely, superframe, subframe, and slot of R-WSANs. Time is divided up into synchronized time slots. Slots are grouped into one superframe, which repeat over time (as illustrated in Figure 3). A slot, typically 10 ms, is long enough to allows exactly one transmission and its associated acknowledgement between a node pair, including encryption/decryption times [6]. Please note that the acknowledgement is used to estimate the TI value of control loops, as we will describe in Section 5.4.

The operation of R-WSAN is broken up into intra-cluster and inter-cluster communications. CHs are responsible for coordination among the end devices within their clusters (intra-cluster communication), and communication with other CHs or GC on behalf of their clusters (inter-cluster communication). The inter-cluster subframe provides the fault-tolerance against possible sensing and actuating link failures.

Each superframe consists with $M_{\mathrm{in}}$ intra-cluster subframes and a single inter-cluster subframe, as shown in Figure 3. The superframe length $T_{\sup}$ must be smaller than the minimum MATI of the control loops to guarantee the control stability. To minimize overhead, the total length of the intra-cluster subframes is long compared to the inter-cluster subframe in the superframe. Hence, the superframe length is

(2) $$\begin{array}{c} T_{\sup}=M_{\mathrm{in}}T_{\mathrm{in}}+T_{\mathrm{out}}\leq \underline{h} \end{array}$$

where $T_{\mathrm{in}}$ is the intra-cluster subframe length, $T_{\mathrm{out}}$ is the inter-cluster subframe length, and $\underline{h}=\min_{1\leq i\leq N_{p}}h_{i}$ is the minimum MATI of plants. Hence, the number of intra-cluster subframes per superframe must satisfy

(3) $$\begin{array}{c} M_{\mathrm{in}}\leq \left\lfloor \frac{\underline{h}-T_{\mathrm{out}}}{T_{\mathrm{in}}}\right\rfloor \;. \end{array}$$

A shorter superframe gives better robustness due to the large number of redundant superframe with respect to MATI $h_{i}$ of plant *i*.

We now present more detailed structure of the intra-cluster and inter-cluster subframes.

#### 5.1.1. Intra-Cluster Subframe

Each cluster contains a CH and several end devices each of which connected to the plant as shown in Figure 1. Each intra-cluster subframe is further divided into a beacon and several data transmission slots for sensing and actuation. The intra-cluster subframe is similar to the frame structure of beacon-enabled IEEE 802.15.4 [42]. After CHs are decided, each CH periodically broadcasts the beacon frame in every intra-cluster subframe $T_{\mathrm{in}}$ to identify its cluster and to synchronize end devices that communicate with it. The beacon message contains the length of intra-cluster subframe, length of superframe, cluster ID, and scheduling decisions to all sensors and actuators of its cluster.

The data transmission slots can be further divided into a contention free period (CFP) and a contention access period (CAP), composed of dedicated TDMA slots and shared slots, respectively. In the CFP, the dedicated bandwidth is used for time-critical sensing and actuating frames. Moreover, the intra-cluster subframe includes the number of shared slots $T_{\mathrm{cap}}$ used by all sensors in the network for transmission, as illustrated in Figure 3. We set the minimum length of CAP ${\underline{T}}_{\mathrm{cap}}$ in each intra-subframe, similar to the IEEE 802.15.4 standard [42]. Please note that CH is always on during CAP for potential senders. During the CAP, the critical plant state is transmitted using a slotted Aloha mechanism. We will discuss more details in Section 5.4.

Communication and computation schedules must be aligned, meaning that measured data (i.e., data from sensors) is forwarded to CH prior to the activation. Since the sensing and actuating links are coupled, we assign the actuating link right after the sensing link slot, as illustrated in Figure 3. Hence, the minimum delay between sensing and actuation is 2 time slots. Given the set of plants $G_{j}$, the minimum length of the intra-cluster subframe to complete the plant schedule of cluster *j* is

(4) $$\begin{array}{c} {\underline{T}}_{\mathrm{in},j}=1+T_{\mathrm{cfp},j}+{\underline{T}}_{\mathrm{cap}} \end{array}$$

where 1 slot is for the beacon transmission, ${\underline{T}}_{\mathrm{cap}}$ is the minimum CAP length, and $T_{\mathrm{cfp},}=\sum_{i\in G_{j}}N_{s,i}+N_{a,i}$ is the dedicated slot length for all cluster members.

We set the intra-cluster length as the largest value of the minimum length of the intra-cluster subframes of all clusters, namely,

(5) $$\begin{array}{c} T_{\mathrm{in}}=\max_{1\leq j\leq N_{ch}}{\underline{T}}_{\mathrm{in},j}={\underline{T}}_{\mathrm{in},j}+T_{\mathrm{rd},j}\;1\leq j\leq N_{ch} \end{array}$$

where $T_{\mathrm{rd},j}$ is the redundant slot of cluster *j* with respect to $T_{\mathrm{in}}$. Hence, a smaller cluster has more redundant slots to use. The redundant slots are used for the slotted random access.

Shorter intra-cluster subframes repeat more often, resulting in lower TI values. The TI of the control loop is basically inversely proportional to the length of the intra-cluster subframe. Similarly, the shorter the subframe, the more often nodes have to wake up to listen or transmit.

#### 5.1.2. Inter-Cluster Subframe

The inter-cluster subframe supports the communication with external GC and other CHs, as shown in Figure 3. Each inter-cluster subframe begins with the configuration updates between GC and CH. Then, the communication with other CHs is followed to provide the fault-tolerance. Each CH aggregates all the sensing data and link PDR in its cluster, and then transmits the compressed data to GC in each inter-cluster subframe. This ensures that GC has a complete picture of the entire plants covered by R-WSAN.

When the intra-cluster performance is significantly degraded even for the short term, this may incur the instability problem of control loops due to the missing sensing and actuating signals. Hence, each CH shares the critical plant state information, the set of the accessible actuators, and its control gains with neighbor CHs. During inter-cluster subframe $T_{\mathrm{out}}$, the concurrent transmissions between neighbor CHs are scheduled in order to minimize the length of the inter-cluster subframe. We must ensure that $T_{\mathrm{out}}\ll T_{\sup}$ to reduce the overhead.

For instance, in Figure 3, after receiving a list of critical control loops of CH 1, CH 2 looks at its list of accessible actuators. In the next slot, CH 2 computes the control signal and transmits it to the corresponding actuator of the critical control loops if it can access it. After the successful transmission of control signals, CH 2 notifies it to the original CH 1, so, it can delete the corresponding plant in the list of the critical control loops.

If GC supports only a single channel as a half-duplex mode, then the length of the inter-cluster subframe must be larger than $2N_{ch}$ for the bi-directional communications between GC and all CHs. Hence, GC can be bottleneck of overall network due to the multiple CHs. We assume that GC concurrently supports the number of channels $C_{gc}$. Then, the minimum length of the inter-cluster subframe becomes $2N_{ch}/\min\left(N_{ch},C_{gc}\right)$ by utilizing the simultaneous communications between GC and CH. To transmit all collected measurements within $T_{\sup}$ to GC, the inter-cluster subframe $T_{\mathrm{out}}$ must be

(6) $$\begin{array}{c} \frac{2N_{ch}}{\min(N_{ch},C_{gc})}\leq T_{\mathrm{out}}\leq \underline{h}\;. \end{array}$$

We apply the maximal set method to maximize the simultaneous transmissions between CHs using different channels for the efficient scheduling [32]. Please note that the concurrent transmission scheduler only considers the primary interference between CHs since GC supports the multiple channels. By considering communications with GC and all CHs, the total inter-cluster length is

(7) $$\begin{array}{c} T_{\mathrm{out}}=2+T_{ch} \end{array}$$

where 2 slots are used for data transfer between GC and CH and $T_{ch}$ is the length of the concurrent transmissions between neighboring CHs. We set the length of the inter-cluster subframe equal to the one of the intra-cluster subframe, $T_{\mathrm{out}}=T_{\mathrm{in}}$ as a default parameter.

### 5.2. Clustering

We propose the centralized clustering method based on the information of both the complete topology of wireless networks and all plants of control systems. GC collects the network PDR graph between end devices and CHs and MATI given by each plant of the network. Starting from the aforementioned information, GC computes the network clusters. Then, GC includes the clustering descriptor which is the plant list that belongs to certain clusters in the following configuration message to announce the clustering. Since each plant has multiple candidate CHs, CHs yielding lower intra-cluster communication cost are preferred.

Periodic re-clustering is triggered every time interval $T_{cu}=M_{cu}T_{\mathrm{in}}$ slots to select new CHs. We set a relatively longer cluster update interval since the reconfiguration cost can be high due to the configuration delay and message losses. At every cluster updating time, CH broadcasts the updated CHs to end devices of its current cluster. When each end device receives the updated CH, it changes the channel hopping sequence in order to receive the beacon message from the new CH. This reconfiguration scheme gives the time delay between releasing event from the previous cluster and updating event from the new cluster.

Our goal of the clustering is to identify a set of clusters which cover the entire plants. Each plant *i*, where $1\leq i\leq N_{p}$, is then mapped to CH *j* where $1\leq j\leq N_{ch}$. Remind that the number of CHs is smaller than the number of plants $N_{ch}\leq N_{p}$. In addition, all end devices attached to the same plant are allocated to the same cluster in order to reduce the delay between sensing and actuating time. We consider both sensing link from plant to CH and actuating link from CH to plant. Due to different locations of sensors and actuators of the plant, the sensing and actuating links do not necessarily symmetric even for the same plant. Hence, we define the connection quality $R_{ij}$ between plant *i* and CH *j* as the product of sensing link PDR $R_{ij}^{s}$ (i.e., from sensor to CH) and actuating link PDR $R_{ji}^{a}$ (i.e., from CH to actuator), namely,

(8) $$\begin{array}{c} R_{ij}=R_{ij}^{s}R_{ji}^{a}\;. \end{array}$$

#### 5.2.1. Clustering Optimization Problem

Now, we formulate the clustering optimization problem to maximize the robustness of the clusters while meeting the clustering constraints. We define the clustering cost as a function of the plant weight of Equation (1) and the connection quality between plant and CH. The association cost of the plant to the cluster increases for the higher weight of the plant $W_{i}$ and lower connection quality $R_{ij}$ between plant *i* to CH *j*. The clustering cost to associate plant *i* to CH *j* is defined as $\frac{W_{i}}{R_{ij}}$. Our objective function is to minimize the maximum clustering cost of all clusters while associating each plant to a cluster of the network. Hence, the clustering assignment problem becomes

(9a)minδ(9b)s.t.∑i=1NpWiRijbij≤δ,1≤j≤Nch(9c)∑j=1Nchbij=1,1≤i≤Np(9d)bij∈{0,1}

where $b_{ij}=1$ if plant *i* is associated with CH *j*, 0 otherwise. Please note that we convert the min-max problem into the constrained minimization problem by introducing additional variable δ. Observe that since the maximum clustering cost depends on the clustering decision of all clusters, the local information is insufficient to achieve the global optimal solution.

The proposed clustering problem is a binary integer programming problem and it can be solved using the well-known branch-and-bound [43]. However, since this problem has a large number of decision variables due to all possible links between plants and CHs even for a small scale network, it can be computationally expensive to solve the optimization problem. Hence, we propose a simple clustering algorithm based on the proposed optimization problem Equation (9).

#### 5.2.2. Clustering Algorithm

GC runs a simple clustering algorithm dependent on the connection quality and the weight of plants. In Algorithm 1, it assigns each plant of the network to a cluster in order to minimize the maximum clustering cost. At the end of algorithm phase, each plant is associated with the cluster based on the binary decision matrix B of size $N_{p}\times N_{ch}$. The element of B is 1 if plant *i* belongs to CH *j*, whereas 0 otherwise.

By considering the maximum connection quality between plant *i* to the set of CHs, the clustering weight of plant *i* is defined as

(10) $$\begin{array}{c} C_{i}=\frac{W_{i}}{\max_{1\leq j\leq N_{ch}}R_{ij}},\;1\leq i\leq N_{p}\;. \end{array}$$

Since the control plant becomes more critical for the greater value of $C_{i}$, GC reorders the set of the plant based on its clustering weight in the descending order. Then, it estimates the clustering cost by adding plant *i* to the set of candidate CHs. The clustering cost vector V is accumulated as adding more plants in Algorithm 1. For a given plant $i^{*}$, GC finds the optimal CH $j^{*}$ to minimize the maximum clustering cost of the network by using a simple search.

**Algorithm 1:** Clustering algorithm of GC. **Input**: Clustering weight of plants with vector of size $N_{p}\times 1$, C where $C_{i}=\frac{W_{i}}{\max_{1\leq j\leq N_{ch}}R_{ij}}$ **Output**: Binary decision matrix of size $N_{p}\times N_{ch}$, B I = Sort (C) ; //
priority index of sorted plant weight in descending order Zero vector of size $N_{ch}\times 1$, V ; //
cumulative cluster cost of CHs( **for**
i←1
**to**
$N_{p}$
**do** 
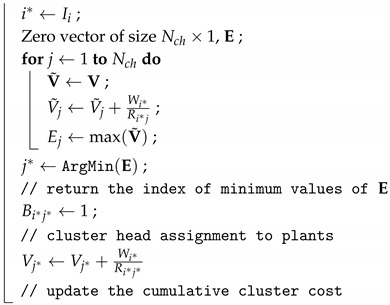


#### 5.2.3. Clustering Validation

**Figure 4:**
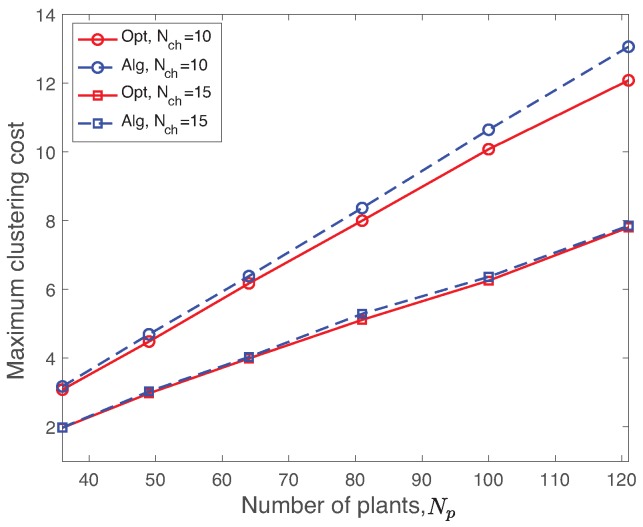
Maximum clustering cost by using the optimal solution and the proposed heuristic algorithm with $N_{ch}=10,15$ as a function of different number of plants $N_{p}=36,\ldots ,121$. The objective value of the proposed clustering algorithm matches well the one using the optimal solutions.

We validate the proposed Algorithm 1 with respect to the ideal solution of the optimization problem Equation (9). Figure 4 shows the maximum clustering cost obtained by the optimization problem and Algorithm 1 with $N_{ch}=10,15$ as a function of different number of plants $N_{p}=36,\ldots ,121$. Please note that we provide the detailed setup of the simulation in Section 6. The maximum clustering cost of the network is proportional to the number of plant while its slope depends on the number of CHs. It is natural that the clustering cost decreases as increasing the number of CHs due to the reduced loads of each cluster. The optimal solution obviously gives the lower bounds of the clustering cost of the network. In Figure 4, the cluster cost difference between the optimal solution and the clustering algorithm increases as increasing the number of plants with $N_{ch}=10$. The mean error of the maximum clustering cost is still less than 10% over the different number of plants $N_{p}\leq 121$.

### 5.3. Scheduling

Once all the end devices are organized into clusters, each CH creates a schedule for the plants in its cluster. In R-WSAN, each link of the intra-cluster is defined as a directed communication between end device and CH in a specific slot. Remind that all plants take place at least two coupled slots for sensing and actuating links, within a single intra-cluster subframe. Each CH builds a TDMA schedule telling each sensor or actuator when it can transmit or receive, respectively. A slot in a subframe is identified by its time offset and its channel offset. CH includes the scheduling descriptor which is the node list that obtains slots in the following beacon to announce the resource allocation. If there is no need to change the scheduling decision, the beacon will only provide synchronization information. The radio of each end device can be turned off until its allocated transmission or reception slot, thus minimizing energy consumption.

#### Slot Scheduling

Each CH runs a local scheduling algorithm based on the connection quality between plant and its CH and MATI given by its cluster members. Remind that each end device and CH cannot transmit and receive at the same time, and it cannot receive from multiple nodes at the same time. Starting from this primary interference constraint, each CH builds the schedule by running Algorithm 2.

The scheduling policy basically relies on the earliest deadline first approach and the connection quality. Each CH computes the scheduling weight of control loops as a function of (i) current control stability margin, namely, current TI margin with respect to MATI (ii) connection quality between plant and CH. Hence, CH *j* defines the scheduling weight of plants $i\in G_{j}$ as

(11) $$\begin{array}{c} P_{i}=\frac{Q_{i}}{R_{ij}},\;\forall i\in G_{j}, \end{array}$$

where $Q_{i}$ is the ratio between current TI $\tau_{i}$ and MATI $h_{i}$ given by


(12) $$\begin{array}{c} Q_{i}=\frac{\tau_{i}}{h_{i}},\;\forall i\in G_{j}\;. \end{array}$$

Each plant of the cluster has to be scheduled more than once in a intra-cluster subframe. We separate the slot scheduling with two parts, namely, $S_{1}$ and $S_{2}$ of Algorithm 2. The default scheduler $S_{1}$ assigns each control loop exactly once in a subframe. The scheduling order of $S_{1}$ is given by the descending order of the scheduling weight vector of Equation (11).

We only activate the additional scheduler $S_{2}$ if any control loops of cluster *j* violate the TI ratio threshold $Q_{i}\geq Q_{thr},\;\forall i\in G_{j}$. CH creates the additional scheduler $S_{2}$ for the available slot resource ${\bar{T}}_{\mathrm{cfp}}-{\underline{T}}_{\mathrm{cfp}}$ where ${\bar{T}}_{\mathrm{cfp}}$ and ${\underline{T}}_{\mathrm{cfp}}$ are the maximum and minimum length of CFP, respectively. Since CH must allocate all sensing and actuating links of cluster members, the minimum length of CFP ${\underline{T}}_{\mathrm{cfp}}$ becomes


(13) $$\begin{array}{cc} {\underline{T}}_{\mathrm{cfp}} & =\sum_{i\in G_{j}}N_{s,i}+N_{a,i}\;. \end{array}$$

Furthermore, the maximum length of CFP ${\bar{T}}_{\mathrm{cfp}}$ is

(14) $$\begin{array}{cc} {\bar{T}}_{\mathrm{cfp}} & =T_{\sup}-{\underline{T}}_{\mathrm{cap}} \end{array}$$

due to the minimum CAP requirement.

**Algorithm 2:** Scheduling algorithm of CH *j*. **Input**: $G_{j},T_{\sup},{\underline{T}}_{\mathrm{cap}},N_{s,i},N_{a,i}$ where $i\in G_{j}$ **Output**: Scheduling vector of plants, S ${\bar{T}}_{\mathrm{cfp}}\leftarrow T_{\sup}-{\underline{T}}_{\mathrm{cap}}$ ; ${\underline{T}}_{\mathrm{cfp}}\leftarrow \sum_{i\in G_{j}}N_{s,i}+N_{a,i}$ ; Vector of size $|G_{j}|\times 1$, Q where $Q_{i}\leftarrow \frac{\tau_{i}}{h_{i}}\;\forall i\in G_{j}$ ; Vector of size $|G_{j}|\times 1$, P where $P_{i}\leftarrow \frac{Q_{i}}{R_{ij}}\;\forall i\in G_{j}$ ; $S_{1}=\mathrm{Sort}\left(P\right)$; //
(array of plant index of sorted P in descending order( **if**
any
*(*$Q\geq Q_{thr}$*)*
**then** 
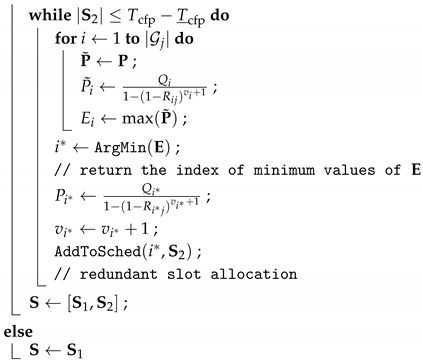


To build $S_{2}$, it basically searches the most critical control loops based on the pre-allocated slots of the subframe. Given the number of pre-allocated slots $v_{i}$, the connection quality of the plant *i* is

(15) $$\begin{array}{c} 1-{\left(1-R_{ij}\right)}^{v_{i}}\;. \end{array}$$

By considering $v_{i}$, the modified scheduling weight of pant *i* is

(16) $$\begin{array}{c} P_{i}=\frac{Q_{i}}{1-{\left(1-R_{ij}\right)}^{v_{i}+1}},\;\forall i\in G_{j} \end{array}$$

instead of Equation (11) for additional scheduler $S_{2}$. Then, Algorithm 2 merges the scheduling sets $S_{1}$ and $S_{2}$.

### 5.4. Critical Control Task

This is one of the novel approaches to improve the control robustness against communication failures of R-WSAN. In Figure 2, the sensing or actuating information can be carried on a set of pre-established multiple neighbor CHs in order to improve the spatial diversity of the network. The spatial diversity of routes allows messages to be delivered to multiple CHs. Each sensor selects the backup CHs based on the link PDR.

**Figure 5:**
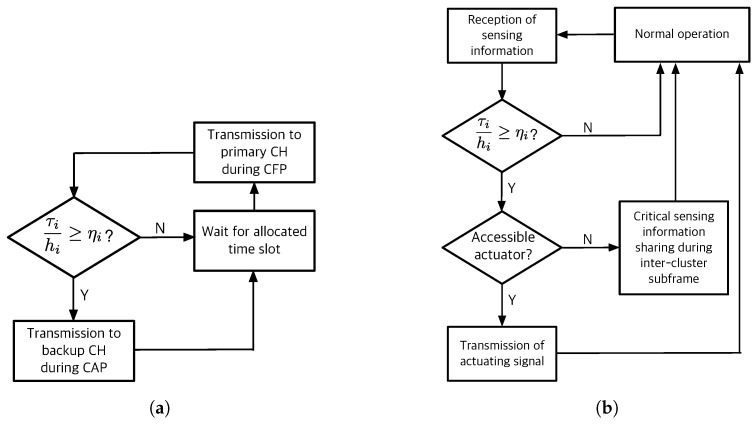
Control task sharing policy of critical loops. (**a**) Access policy to backup cluster head of critical sensor node; (**b**) Backup cluster head operation for control task sharing.

Figure 5 illustrates the basic operation of sensor and backup CHs to provide the control robustness of critical loops. When each sensor sends the sensing information to CH, CH confirms its receipt by sending an acknowledgement frame. Hence, each sensor easily tracks the current TI. If the TI of control loop is closer to the critical MATI due to consecutive losses, the sensor transmits its plant state to pre-established multiple CHs as the backup controllers. Each node activates the slotted Aloha mechanism to forward the critical plant state information if $\frac{\tau_{i}}{h_{i}}>\eta_{i}$ where the activation threshold is $\eta_{i}=\frac{2T_{\sup}}{h_{i}}$. Since many sensors may contend to transmit its plant state due to correlated channel, it transmits the measurements with probability $\rho_{c}$ to avoid the packet collision.

By using the intra-cluster subframe and channel hopping mechanism, each sensor knows the random access mode of each cluster and receiving channel even if it does not associate to other clusters. Since each intra-cluster subframe includes the minimum CAP, as shown in Figure 3, each sensor computes the shared slots of each backup CH. Furthermore, they calculate the receiving channel of neighbor CHs in each time slot because the channel hopping only requires the global slot counter and CH ID. This mechanism does not require any heavy communication and computation overhead. CH is only required to turn on their radios and await a potential packet from the sensors without any extra negotiation. Even if the received sensing information does not associate to its cluster, CH computes the control signal of the critical control plant if it can access the actuator and transmit it right after the end of CAP, as illustrated in Figure 3 and Figure 5. Otherwise, it also shares the critical plant state with other CHs during the inter-cluster subframe.

The random access mechanism of each sensor can improve the network robustness since it can transmit the critical sensing information to multiple CHs for extended shared slots rather than the relatively short CAP of its primary CH. Furthermore, the critical sensor transmits the measurements to a randomly selected CH out of the multiple candidates of CHs. Hence, the sensing traffic is distributed over different neighbor CHs. The actual contention level is reduced since each cluster operates at different channels. Hence, the proposed control task sharing approach provides the fast adaptation of critical control loops through the simple slotted random access and the inter-cluster communication.

## 6. Performance Evaluation

In this section, we evaluate the performance of R-WSAN via discrete event-based simulations using Matlab. We assume that plants are uniformly dispersed into a field with dimensions 100 m × 100 m. Sensors and actuators are randomly placed around each plant with circle radius 3 m. The default parameters are given in Table 1 unless it is specified in each simulation. We consider a reasonable number of end devices between 72 and 242 while each plant has $N_{s}=N_{a}=1$ to simplify the simulation setup. Many practical control systems consist of a single sensor and a single actuator attached to a plant such as flow level control [44], vibration control [45], and multi-agent robot control [46]. Please note that the number of nodes for the factory automation and power system protection are around 100 [3]. We assume that the MAD is equal to the MATI value. We obtain the simulation results out of 10 experimental runs of $10^{5}$ slots each.

We compare the proposed R-WSAN protocol to a traditional centralized approach that is suitable for the practical industrial automation [5]. In the centralized approach, all sensors transmit the plant state to GC through CH. Then, GC runs the control algorithm and forwards the control signal to the corresponding actuator as a centralized controller. In addition, it does not allow the task sharing between CHs, since GC is the only one controller of the network. Hence, we slightly modify the intra-cluster subframe with additional 2 slots for the communications between CH and GC at the beginning of the intra-cluster subframe. The centralized approach reduces the overhead since it does not require CAP, $T_{\mathrm{cap}}=0$ of the intra-cluster subframe and the inter-cluster subframe $T_{\mathrm{out}}=0$ of the superframce. Furthermore, we assume the number of supporting channels of GC is proportional to the number of clusters. This implies that the considered centralized protocol provides a good baseline of the performance since the centralized one gives much worse feedback delay between sensing and actuation and TI performance due to the limited number of supporting channels in practice.

The proposed R-WSAN is validated through different link models with homogeneous, heterogeneous, and burst links. The link PDR is exponentially decreasing for the transitional distance regions in both theory and practice [47]. Based on the link model [47], the link PDR is

(17) $$\begin{array}{c} \mathrm{PDR}=\max\left(1-\exp(-\alpha |d_{\max}-d|),0\right) \end{array}$$

where α∈[0,1] is the gain exponent dependent on the modulation, coding, and channel condition, *d* is the distance between transmitter and receiver, $d_{\max}$ is the maximum allowable distance *d* beyond which the PDR becomes zero. The link PDR is bounded between 0 and 1 for $0\leq d\leq d_{\max}$ and α∈[0,1]. The higher α, the better the link PDR.

**Figure 6:**
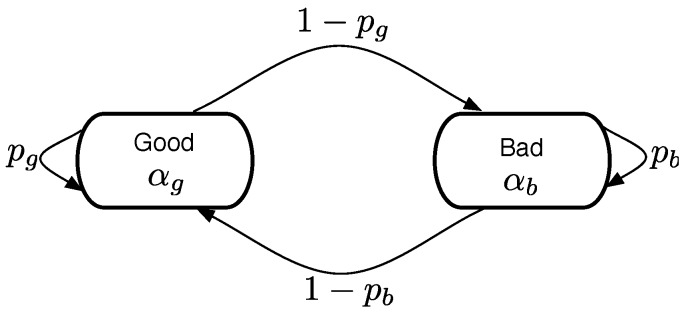
Gilbert-Elliott burst link model.

We define the link model parameters $\alpha_{s},\alpha_{ch},\alpha_{gc}$ dependent on transmitter types of sensor, CH, and GC, respectively. The homogeneous link sets the equal gain exponent for all links of the network, but the heterogeneous link assigns the higher gain exponents for GC and CH since these nodes possibly have better radio transceiver. Furthermore, since most wireless links show the burst behavior [48], we model the burst link using a well-known Gilbert-Elliott model, as illustrated in Figure 6. Table 1 shows the model parameters of different links.

In this section, we analyze the performance of our R-WSAN under both transient and stationary conditions by Monte Carlo simulations. We compare R-WSAN to the centralized protocol in terms of: (i) intra-cluster failure (ii) inter-cluster failure (iii) number of plants (iv) number of CHs, and (v) heterogeneous control systems. We also present the performance metric based on the characteristics of feedback delay and TI.

### 6.1. Effect of Intra-Cluster Failure

**Figure 7:**
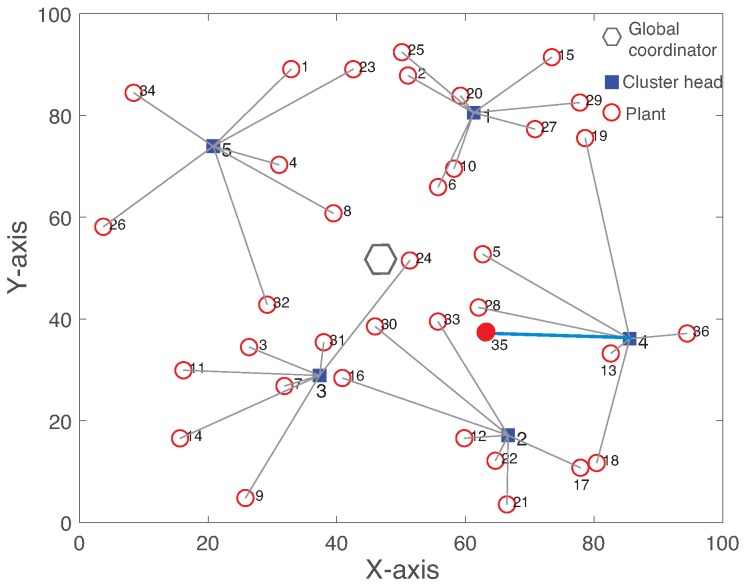
Clustered network topology with $N_{p}=36$ and $N_{ch}=5$. The hexagon, rectangle, and circle represent global coordinator, cluster head, and plant, respectively. The link between plant and cluster head indicates the associated cluster. The intra-cluster sensing link between plant 35 and cluster head 4 is failed at 110 time slots.

We analyze the effect of the intra-cluster communication failure on both R-WSAN and the centralized approach. Figure 7 shows the network topology with the number of plant $N_{p}=36$ and number of CHs $N_{ch}=5$. The hexagon, rectangle, and circle represent GC, CH, and plant, respectively. The link between plant and CH indicates the associated cluster obtained by Algorithm 1. In addition, each plant has backup CHs to improve the operation robustness. We simulate a scenario where the initial topology is changed due to the sensing link failure from sensor of plant 35 to its CH 4 at 110 time slots, as shown in Figure 7.

**Figure 8:**
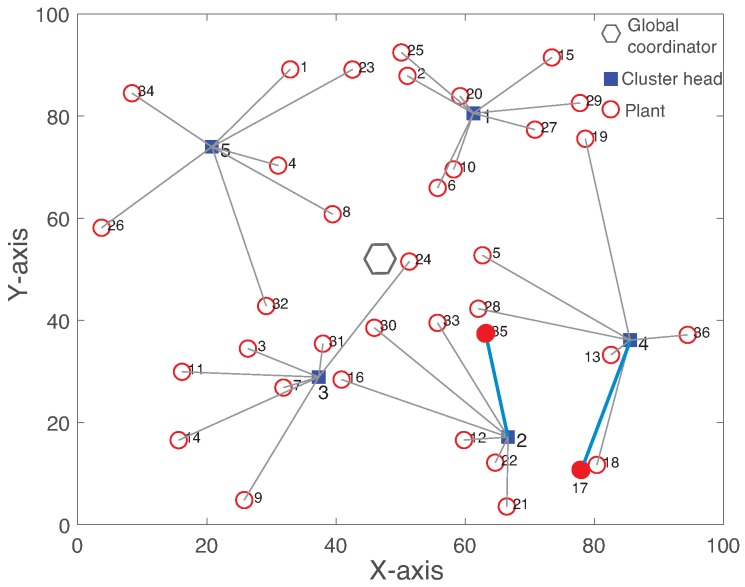
Reconfigured network clustering after the intra-cluster link failure between plant 35 and cluster head 4.

Figure 8 illustrates the reconfigured cluster by Algorithm 1 to minimize the maximum clustering cost of the network. After the cluster reconfiguration by GC, plant 35 changes its cluster to new CH 2 instead of CH 4 due to the sensing link failure. By comparing Figure 7 and Figure 8, while plant 35 changes its cluster, plant 17 also switches to CH 4 from CH 2. Hence, CH 2 and 4 exchange their plant members to minimize the maximum clustering cost of the network. Both R-WSAN and the centralized one activate the clustering algorithm in a fixed time interval due to the configuration overhead and the possible faults. It is practically difficult to achieve the deterministic reconfiguration delay due to configuration message losses from GC to end devices through the clusters. In fact, even if the cluster reconfiguration is successful, this results in a substantial disruption of the sensing and actuating updates of the control systems.

**Figure 9:**
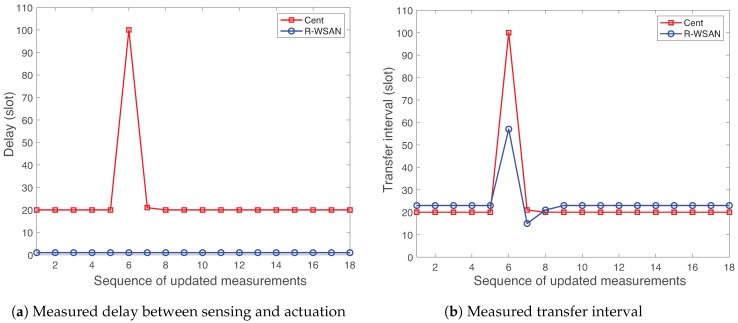
Effect of intra-cluster failure of R-WSAN and the centralized protocol as a function of sequence of updated measurements. R-WSAN provides the robust delay and transfer interval performance against the intra-cluster link failure.

Figure 9 presents the measured delay between sensing and actuation and TI of plant 35 using R-WSAN versus the centralized protocol. In the figure, note that “Cent” refers the centralized protocol. The link failure rapidly deteriorates the delay and TI performance. In Figure 9a, the delay of R-WSAN is constant since CH directly forwards the control signal to actuator based on the received sensing signal. However, the centralized protocol gives higher delay due to end-to-end multi-hop delay from sensing to actuation. In fact, at 110 time slots, the sensing link failure significantly increases the feedback delay and it is resolved only after the successful cluster reconfiguration in the centralized scheme.

In Figure 9b, we clearly observe the effect of link failure in terms of the TI metric. Before the link failure, the centralized protocol gives sightly lower TI than the one of R-WSAN due to the shorter length of the supeframe, which is different from the delay behavior. The centralized protocol provides the similar delay and TI performance since the sensing and actuating links are separately activated for two sequential superframes. Remind that the control signal is only computed at GC after receiving the plant state. Hence, it is not feasible to reduce the feedback delay unless we include additional slots for GC and CH communications in each sensing and actuating link. This will significantly increase the operation overhead. While the sensing link failure significantly degrades the TI performance until the cluster reconfiguration, its effect is much lower for the R-WSAN protocol. The main robust benefit of R-WSAN is due to the efficient task sharing technique to handle the sensing link failure. The sensor of plant 35 activates the critical task sharing policy and accesses the backup CH. Then, it forwards its plant state to the new backup CH during its CAP. Hence, there is a fundamental tradeoff between robustness and average performance by utilizing the fault-tolerant mechanism.

Now, we illustrate the effect of the intra-cluster failure on the control performance. We consider a linear time invariant system where every sensing and actuating links are closed over the wireless network [49]. The plant state-space model [49] is

(18) $$\begin{array}{c} \dot{x}\left(t\right)=Ax\left(t\right)+Bu\left(t\right) \end{array}$$

where

(19) $$\begin{array}{cc} A & =\left[\begin{array}{cc} 0 & 1\\ 0 & -0.1 \end{array}\right],\;B=\left[\begin{array}{c} 0\\ 0.1 \end{array}\right] \end{array}$$

and the state feedback

(20) $$\begin{array}{c} u(t)=-[3.75\;\;11.5]\;x(t) \end{array}$$

and the initial state

(21) $$\begin{array}{c} x_{0}=\left[\begin{array}{c} 2\\ 2 \end{array}\right]\;. \end{array}$$

This example is used to compare multiple overapproximation techniques when analyzing the stability of a control system [49]. In actuation, we apply one of the most popular approaches called the logical zero-order hold mechanism to discard disordered messages [13]. In this mechanism, the latest message is kept and old messages are discarded based on the time stamp of the received control messages. The simulation results of the communication performance are taken as an input to the control system model.

**Figure 10:**
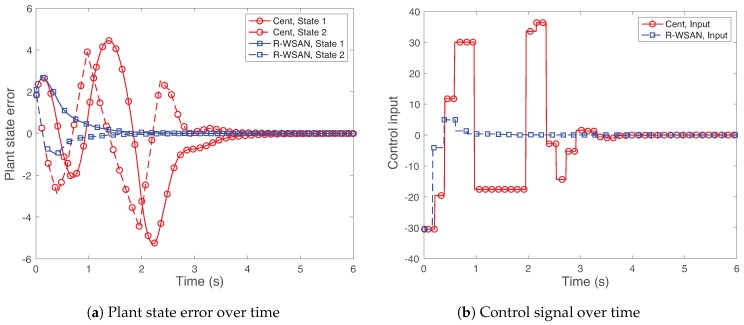
Step response of the control systems against the intra-cluster failure. R-WSAN provides the reliable control performance without any significant overshoot.

We compare the step response of the control system by plotting the output error of Figure 10a and control signal of Figure 10b using R-WSAN and the centralized protocol. Observe that the step response of R-WSAN performs well without any significant overshot. It shows how the R-WSAN adaptation to unplanned changes of the link quality keeps the system response similar to that for the initial topology. On the other hand, the re-clustering algorithm of the centralized protocol results in a system response that rapidly deteriorates in both plant state error and control input. The control system performs inefficiently, due to increase in end-to-end delay from sensors to actuators through GC. Even though the centralized protocol stabilizes the control system, it significantly increases the rise time and the settling time with the larger overshot due to the poor feedback delay and TI performance. Moreover, the control input is highly oscillating for the longer time, as shown in Figure 10b.

### 6.2. Effect of Inter-Cluster Failure

**Figure 11:**
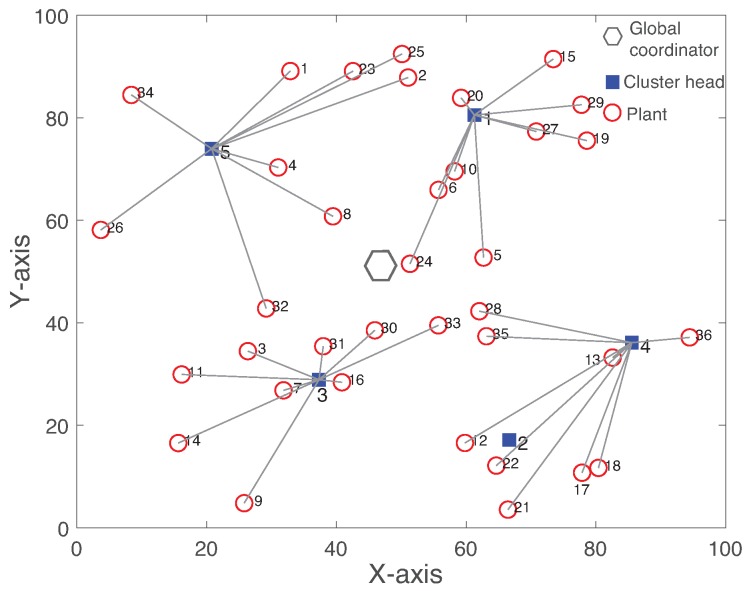
Reconfigured network clustering of the centralized approach after the inter-cluster link failure between global coordinator and cluster head 2.

Now, we analyze the effect of the inter-cluster link failure on the network performance. In Figure 7, let us consider the link failure between GC and CH 2 at 110 time slots. Figure 11 illustrates the reconfigured cluster of the centralized protocol. GC distributes all cluster members of CH 2 to other neighbor clusters. Since our objective of the clustering is to minimize the maximum clustering cost, the inter-cluster failure affects entire clusters of the network with respect to the one of the intra-cluster failure of Figure 8. R-WSAN does not reconfigure the cluster since CH 2 provides the distributed controller to all plant members and receives the central information through the inter-cluster subframe.

**Figure 12:**
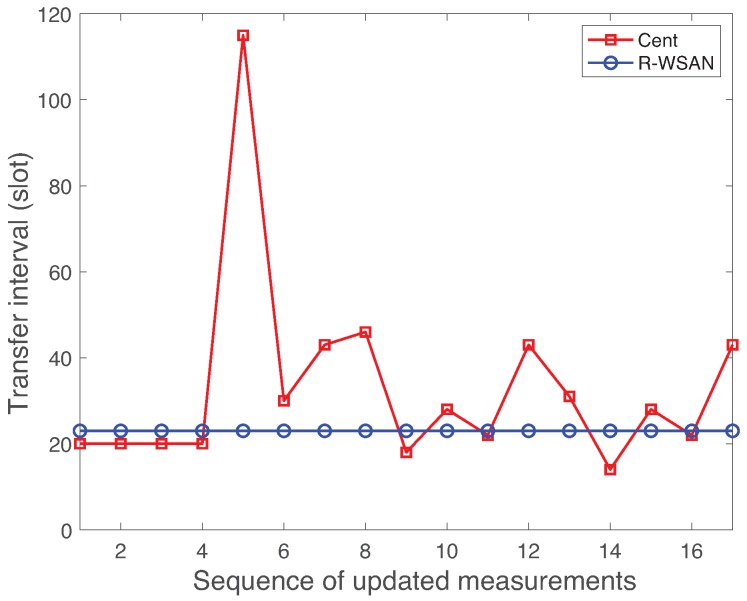
Measured transfer interval of plant 17 using R-WSAN and the centralized protocol as a function of sequence of updated measurements. The inter-cluster link is failed between global coordinator and cluster head 2.

Figure 12 presents the TI measurements of plant 17 using R-WSAN versus the centralized protocol for the inter-cluster failure. We select plant 17 since it is one of the plants of CH 2 as a case study. It clearly shows the significant performance degradation of the centralized approach while the R-WSAN provides the robust performance. Since CH directly controls the plant and shares its plant state with neighbor CHs, R-WSAN provides the stable performance against the inter-cluster link failure. However, the inter-cluster link failure gives catastrophic effect for the centralized controller since GC must change all plant members of the failed cluster. Furthermore, it degrades the overall system performance even if it successfully reconfigures the clusters due to higher re-clustering cost of the network.

### 6.3. Histogram of Delay and Transfer Interval

**Figure 13:**
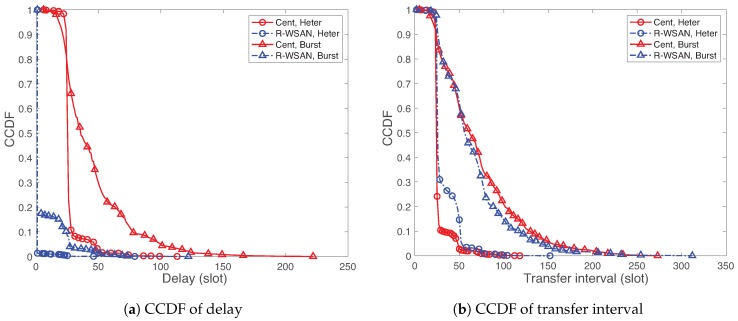
CCDF of delay and transfer interval using R-WSAN and the centralized protocol with different heterogeneous and burst links. Please note that the solid line and dotted line report the performance using the centralized protocol and R-WSAN, respectively, unless it is specified in each simulation. R-WSAN provides the significantly better feedback delay performance with respect to the one using the centralized protocol.

We investigate the detailed delay and TI measurements and define the main performance metrics. Figure 13 shows the complementary cumulative density function (CCDF) of measured delay and TI using R-WSAN versus the centralized protocol with $N_{p}=100$, $N_{ch}=10$ and different heterogeneous and burst link models. Please note that the solid line and dotted line report the performance using the centralized protocol and R-WSAN, respectively, unless it is specified in each simulation. In addition, “Heter” and “Burst” refer the heterogeneous and burst links, respectively. Obviously, the lower is the CCDF, the better is the performance. Both figures shows the worse delay and TI measurements of the burst link than the ones of the heterogeneous link.

In Figure 13a, we clearly observe the significant gap of delay CCDFs between R-WSAN and the centralized protocol. R-WSAN gives a significantly lower delay than the centralized one due to the tightly assigned sensing and actuating slots for the distributed controller. In fact, most delays of R-WSAN are around 2 slots. While the different link models affect the delay of the centralized protocol, its effect is limited for R-WSAN. Hence, R-WSAN provides more robust delay performance than the one of the centralized protocol.

On the other hand, the TI CCDFs between R-WSAN and the centralized protocol are similar to those shown in Figure 13b. The TI CCDF of R-WSAN is slightly higher than the one of the centralized one for the heterogeneous link. However, the burst link gives the opposite results.

Based on the CCDFs of delay and TI, we observe the significantly different behaviors over various percentile ranges. The robustness evaluation is not trivial since we consider the rare events of the simulations such as high delay and TI performance rather than the average performance. To quantify the robustness, we mainly use the 95-th percentile of delay and TI, as a default performance metric in this paper.

### 6.4. Effect of Number of Plants

**Figure 14:**
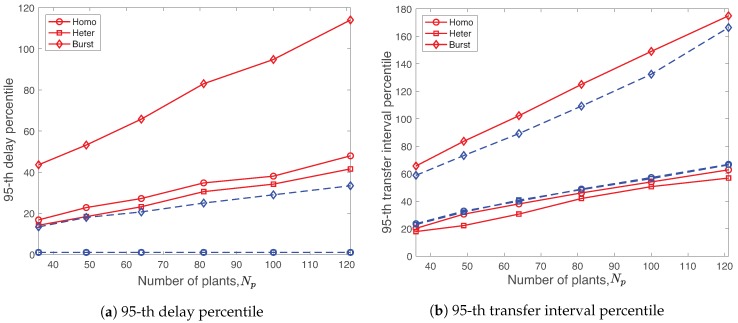
Delay and transfer interval percentiles using R-WSAN and the centralized protocol with homogeneous, heterogeneous, and burst links as a function of different number of plants $N_{p}=36,\ldots ,121$. R-WSAN provides the significantly lower feedback delay than the one using the centralized protocol. Furthermore, the transfer interval performance of R-WSAN is robust over burst links.

We vary the number of plants from 36 to 121 to study how the protocol works with low to high plant density. Figure 14 illustrates the 95-th percentile values of delay and TI of R-WSAN and the centralized protocol with $N_{ch}=10$ and various homogeneous, heterogeneous, and burst links as a function of different number of plants $N_{p}=36,\ldots ,121$. In general, the delay and TI percentiles of both R-WSAN and the centralized protocol increase as the number of plants increases due to the longer superframe length.

Let us first consider the delay and TI performance under the homogeneous and heterogeneous links. In Figure 14a,b, even though the heterogeneous link improves the delay and TI percentiles of the centralized protocol than the one of the homogeneous link, its benefit is minor. The delay and TI performance of R-WSAN are similar for different homogeneous and heterogeneous links by using the distributed controller. For both homogeneous and heterogeneous links, even though the TI percentile of R-WSAN is slightly higher than the centralized one in Figure 14b, the delay percentile of R-WSAN is significantly better, as shown in Figure 14a. Please note that the 95-th delay percentiles of both homogeneous and heterogeneous links are around 2 slots for R-WSAN. Furthermore, R-WSAN provides the reliable delay and TI percentiles as increasing number of plants for both homogeneous and heterogeneous links.

On the other hands, the burst link significantly affects the overall delay and TI performance as shown in Figure 14 for both protocols. We clearly observe the lower delay and TI percentiles of R-WSAN than the one of the centralized one. It is natural that the percentile values of delay and TI of the centralized protocol is more vulnerable due to the multi-hop communication between end device and centralized controller. Hence, R-WSAN provides the significantly better robustness against the burst link with the minor overhead.

### 6.5. Effect of Number of CHs

**Figure 15:**
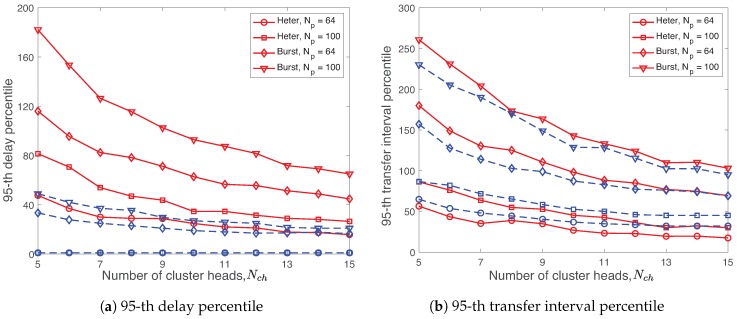
Delay and transfer interval percentiles using R-WSAN and the centralized protocol with $N_{p}=64,100$ and heterogeneous and burst links as a function of different number of cluster heads $N_{ch}=5,\ldots ,15$. Increasing the number of cluster heads does not significantly improve the feedback delay and transfer interval for $N_{ch}>10$.

With a similar way, we investigate the effect of the number of CHs for the R-WSAN protocol. Figure 15 illustrates the 95-th delay and TI percentiles of R-WSAN and the centralized protocol with $N_{p}=64,100$ and different heterogeneous and burst links as a function of various number of CHs $N_{ch}=5,\ldots ,15$. The larger the number of CHs, the smaller the number of associated members per cluster of the network. We observe the similarly effect of the burst link on the delay and TI percentiles of both R-WSAN and the centralized protocol. The number of CHs is not critical for the delay percentile of the R-WSAN protocol for $N_{ch}\geq 9$ in Figure 15a.

One interesting observation is that increasing the number of CHs does not significantly improve the delay and TI percentiles of both protocols under different links. Even though the TI percentile of both R-WSAN and the centralized protocol is improved for $5\leq N_{ch}\leq 10$ when $N_{p}=100$, the performance benefit becomes minor for the large number of CHs $N_{ch}>10$. This is not surprising since the superframe structure includes the control overhead independently from the number of plants.

### 6.6. Effect of Heterogeneous Plant

We investigate the adaptability of the R-WSAN protocol to three different plant classes, namely, high, middle, low priority classes, with different MATIs, h=90,120,150 slots, respectively. We set the ratio of the number of plants between three classes as 0.2,0.3,0.5 for high, middle, low priority classes, respectively.

To quantify the robust performance with heterogeneous requirements, we define the redundancy gain of class *i* as

(22) $$\begin{array}{c} \gamma_{i}=\frac{h_{i}-{\tilde{\tau }}_{i}}{h_{i}} \end{array}$$

where $h_{i}$ is the MATI of class *i* and ${\tilde{\tau }}_{i}$ is the 95-th TI percentile of class *i*. Hence, the higher is the redundancy gain, the better is the TI performance with respect to MATI. In Equation (22), we also define the delay redundancy gain by replacing the TI and MATI values with the delay and MAD, respectively.

**Figure 16:**
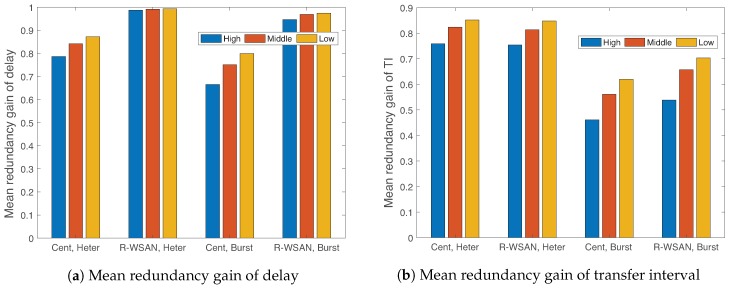
Mean redundancy gain of delay and transfer interval of three classes using R-WSAN and the centralized protocol with different heterogeneous and burst links. R-WSAN provides the robust delay and transfer interval performance for heterogeneous control requirements.

Figure 16 compares the redundancy gains of delay and TI of three classes using R-WSAN versus the centralized protocol with $N_{p}=64$, $N_{ch}=10$, and different heterogeneous and burst links. By considering the different MATIs, the clustering algorithm minimizes the maximum clustering cost of the network. The number of plant members is reduced when the critical plant is assigned to the cluster in order to balance the clustering cost of the network. Both solutions meet the MAD and MATI requirements since the redundancy gains of delay and TI are positive.

In Figure 16a, we clearly observe the significant delay redundancy gain of the R-WSAN protocol compared to the one of the centralized protocol in both heterogeneous and burst links. On the other hand, in Figure 16b, R-WSAN provides the comparable redundancy gain of TI for the heterogeneous link. However, R-WSAN can achieve around 15% more gains of the TI performance with respect to the centralized one under the burst link. In Figure 16a,b, the redundancy gains of delay and TI decrease as increasing the priority of classes due to Equation (22).

**Figure 17:**
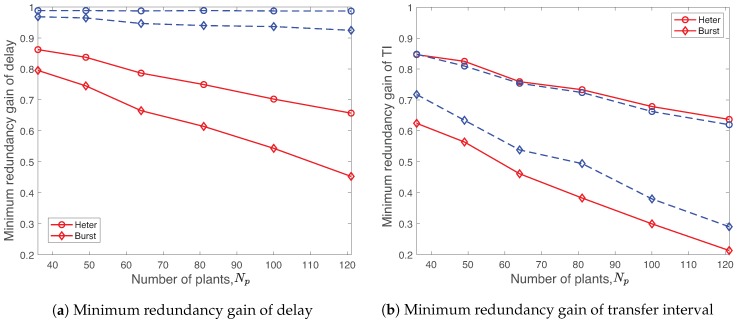
Minimum redundancy gains of delay and transfer interval of three classes using R-WSAN and the centralized protocol with heterogeneous and burst links as a function of different number of plants $N_{p}=36,\ldots ,121$. R-WSAN efficiently adapts the network resources for heterogeneous control requirements.

Figure 17 presents the minimum redundancy gains of delay and TI of three classes obtained by R-WSAN and the centralized protocol with heterogeneous and burst links as a function of different number of plants $N_{p}=36,\ldots ,121$. Results indicate that the R-WSAN protocol outperforms the centralized one in terms of the delay and TI redundancy gains for most of the varying parameters and link conditions. For the heterogeneous link, Figure 17b also shows that the TI gains of both R-WSAN and the centralized one are comparable. Hence, the R-WSAN protocol effectively handles the heterogeneous requirements of the control systems.

## 7. Conclusions

In this paper, we proposed the R-WSAN protocol to maintain the control stability against the network faults such as node and link failures. The proposed joint design approach combines the distributed controller of control systems and the clustering, scheduling, and control task sharing scheme of wireless networks to guarantee the control stability constraint. Specifically, the cluster heads of R-WSAN share the critical control tasks by using the slotted random access and the inter-cluster communication. Simulation results showed that R-WSAN ensures the control stability of multiple plants while enhancing the resilience to the network faults for sensing and actuation with negligible overhead. In addition, we showed that even if the intra-cluster and inter-cluster failures occur, R-WSAN provides the robust control performance by using the efficient control task sharing scheme. Our results highlight the effectiveness of the joint design of wireless networks and control systems in WNCSs. Future works include the practical implementation of R-WSAN using Zolertia sensors [50] based on the specifications of the IEEE 802.15.4e standard [23]. The optimization algorithms are successfully implemented and evaluated through embedded wireless nodes for control systems [16,32].

## Figures and Tables

**Table 1:** Default simulation parameters used in the paper. We consider three different link models, namely, homogeneous, heterogeneous, and burst links.

Link Model	Meaning	Value
	Deployed range	100 m × 100 m
	Number of plants, $N_{p}$	$36\leq N_{p}\leq 121$
	Number of sensors of each plant, $N_{s}$	1
	Number of actuators of each plant, $N_{a}$	1
	Time slot duration	10 ms
	Clustering update interval, $M_{cu}$	5
	Number of intra-cluster subframe per superframe, $M_{\mathrm{in}}$	5
	Minimum length of CAP, ${\underline{T}}_{\mathrm{cap}}$	3 slots
	Threshold to activate additional scheduler $S_{2}$, $Q_{thr}$	0.5
	Channel access probability, $\rho_{c}$	0.5
	MATI, MAD	120 slots
Homogeneous link	Gain exponent of sensor, $\alpha_{s}$	0.1
	Gain exponent of CH, $\alpha_{ch}$	0.1
	Gain exponent of GC, $\alpha_{gc}$	0.1
Heterogeneous link	Gain exponent of sensor, $\alpha_{s}$	0.1
	Gain exponent of CH, $\alpha_{ch}$	0.2
	Gain exponent of GC, $\alpha_{gc}$	0.3
Burst link	Gain exponent of sensor at good state, $\alpha_{s,g}$	0.1
	Gain exponent of sensor at bad state, $\alpha_{s,b}$	0.01
	Gain exponent of CH at good state, $\alpha_{ch,g}$	0.2
	Gain exponent of CH at bad state, $\alpha_{ch,b}$	0.01
	Gain exponent of GC at good state, $\alpha_{gc,g}$	0.3
	Gain exponent of GC at bad state, $\alpha_{gc,b}$	0.01
	Transitional probability, $p_{g}=p_{b}$	0.8
